# Radiographic joint damage in rheumatoid arthritis is associated with differences in cartilage turnover and can be predicted by serum biomarkers: an evaluation from 1 to 4 years after diagnosis

**DOI:** 10.1186/ar1882

**Published:** 2006-01-10

**Authors:** SMM Verstappen, AR Poole, M Ionescu, LE King, M Abrahamowicz, DM Hofman, JWJ Bijlsma, FPJG Lafeber

**Affiliations:** 1 Department of Rheumatology and Clinical Immunology, University Medical Center Utrecht, The Netherlands; 2 Joint Disease Laboratory, Shriners Hospital for Children, Departments of Surgery and Medicine, McGill University, Montreal, Canada; 3 IBEX Pharmaceuticals, Montreal, Canada; 4 Department of Epidemiology and Biostatistics, McGill University, Montreal, Canada; 5 Department of Rheumatology, Hilversum Hospital, Hilversum, The Netherlands

## Abstract

**Introduction:**

The objective of this study was to determine whether serum biomarkers for degradation and synthesis of the extracellular matrix of cartilage are associated with, and can predict, radiographic damage in patients with rheumatoid arthritis (RA).

**Methods:**

Clinical and radiographic data of 87 RA patients were recorded 1 year after disease onset and then annually up to four years. Serum concentrations of four cartilage biomarkers were determined at these time points: a neoepitope formed by collagenase cleavage of type II collagen (C2C), a neoepitope formed by collagenase cleavage of type II collagen as well as type I collagen (C1,2C), a carboxy propeptide of type II procollagen formed during synthesis (CPII), and a cartilage proteoglycan aggrecan turnover epitope (CS846-epitope). Biomarker concentrations between patients with rapid radiographic progression (>7.3 Sharp/van der Heijde units per year) and those with slow radiographic progression (<2.3 units per year) were compared. In addition, we evaluated the long-term and short-term predictive value of each biomarker for progression of radiographic damage.

**Results:**

Patients with rapid radiographic progression had higher C2C, higher C1,2C, and higher CS846-epitope levels than slow progressors. CPII levels showed no differences. Most importantly, the long-term radiographic progression for C2C, for C1,2C, and for CS846-epitope can be predicted by the biomarker value at year 1 after disease onset. C2C was also a predictor for joint space narrowing and annual radiographic damage during the subsequent year.

**Conclusion:**

This study shows that the concentration of serum biomarkers of cartilage collagen breakdown and proteoglycan turnover, but not of collagen synthesis, are related to joint destruction in RA. The use of these biomarkers may be of value when studying progression of joint damage in patients with RA.

## Introduction

Rheumatoid arthritis (RA) is a chronic inflammatory disease characterized by joint destruction. Inflammation of the synovial tissue causes damage to articular cartilage and subchondral bone of the joints [1]. In established RA, radiographs reveal joint space narrowing as a result of cartilage loss and characteristic erosions of bone. Different radiographic scoring methods, such as those of Sharp/van der Heijde [2] or Larsen [3], are used to determine damage in these joints.

The progression of joint damage as measured on radiographs differs significantly between patients. In some cases the progression of joint damage is very slow, whereas in other cases extensive destruction can occur within a few years after disease onset [4]. Radiographs only reveal gross anatomical changes. It usually takes at least one year before significant changes in joint damage can be observed. This delay means there is a need for more sensitive measures that can detect the process of bone and cartilage damage, which may therefore be predictive of radiographically assessed progression of joint damage.

As a result of cartilage damage, key components of the extracellular matrix are lost. This usually results in increased matrix turnover involving increased matrix synthesis in an attempt to replace essential structural components. These turnover products enter body fluids as 'biomarkers', where they can now be detected by sensitive immunoassays in accessible fluids such as serum and urine [5-9]. These biomarker assays may be of use in distinguishing RA patients with rapid progressive and slow progressive joint damage, and may help to identify the severity of joint disease resulting in radiographic damage.

It has been shown in previous studies that a neoepitope marker for degradation of type II collagen in cartilage (C2C), generated by collagenases, is increased in the serum and urine of patients with RA [10]. A related neoepitope COL2-3/4C_Short _(a marker for degradation of type I collagen and type II collagen in cartilage [C1,2C]) can be detected in type II collagen as well as in type I collagen [11]. Elevated levels of urine CTX-II (a c-telopeptide degradation product of type II collagen) have been demonstrated to be associated with increased radiographic damage in RA [12]. Levels of an epitope present in chondroitin sulfate of the cartilage proteoglycan aggrecan (a marker for aggrecan turnover in cartilage [CS846-epitope]) are elevated in serum in chronic RA, although the levels are depressed in rapid progressive RA [13]. The CS846-epitope is found only on the largest aggrecan molecules [14]. Increased serum levels may therefore reflect increased turnover of newly formed matrix, which is normally not seen in adult cartilage. Levels of a marker for synthesis of the procollagen of type II collagen cartilage (CPII), the C-propeptide of type II cartilage collagen, are elevated in RA patients [13,15]. CPII is a marker of increased collagen type II synthesis, and higher values indicate an attempt to repair.

Although the relationship between these markers and joint damage in RA is important to establish, it is more important to determine whether these markers have the capacity to predict the development of joint damage, and hence disease progression. Validation of existing and new biomarkers in this respect is therefore very important in more than just one study before these markers can be used as an additional prognostic measurement in daily practice. More recently, ELISA assays for C1,2C, for C2C, for CS846-epitope, and for CPII have become commercially available. The goal of the present study was therefore to determine whether there is a relationship between concentrations of these serum biomarker and joint damage as well as the progression of joint damage.

Evaluations of this kind should preferably be performed in a cohort of patients with early RA, for whom both radiographic and clinical data have been assessed in detail during the course of disease. We have studied a cohort of RA patients followed from the onset of disease (the Utrecht Rheumatoid Arthritis Cohort [16,17]) for which annual clinical and radiographic data were available. For this study we determined serum biomarker levels annually 1 year from onset and for the subsequent three years.

## Patients and methods

### Patients

From 1990 to 1998, patients with recent onset of RA according to the 1987 American College of Rheumatology revised criteria [18] and disease duration <1 year were recruited from six rheumatology departments in the region of Utrecht, The Netherlands, to participate in a randomized, prospective clinical trial. At entry to the trial, patients were randomly assigned to a group for early initiation with disease-modifying antirheumatic drug therapy (to either start with methotrexate, intramuscular gold, or hydroxychloroquine) or to a group for delayed initiation of disease-modifying antirheumatic drugs.

All patients except two used disease-modifying antirheumatic drugs for some period of time during the follow-up. The protocol of this trial stated that the use of glucocorticoids should be avoided as much as possible during the study period. Only six patients had to use glucocorticoids within the first year after diagnosis. The treatment strategy was decided after 2 years by the treating rheumatologist. There were no statistically significant differences in any of the four biomarker levels between the four treatment groups at 1 year. This study was approved by the medical ethical committees of all participating hospitals, and all patients gave written informed consent before entering the study. The study design and the results of this 2-year treatment trial have been described extensively in previous reports [17,19].

### Study design

To avoid unknown effects directly associated with the initial response to treatment, the evaluation of biomarkers commenced 1 year after the initiation of treatment and continued for a period of 3 years thereafter. Clinical variables were therefore assessed, radiographs taken, and blood samples collected and stored at 1, 2, 3 and 4 years after diagnosis. For this study we used all samples that were still available from the cohort described and those samples that had never been thawed before.

### Clinical variables

The following clinical variables were assessed: erythrocyte sedimentation rate (ESR, mm/h^1st^), morning stiffness (range 0–720 minutes), visual analogue scale for pain (range, 0–100 mm; 100 mm = worst score), visual analogue scale for general well-being (range 0–100 mm; 100 mm = worst score), functional disability (Health Assessment Questionnaire, Dutch version) (range, 0–3; 3 = worse functional ability), rheumatoid factor (positive/negative), either by means of the Waaler Rose test (positive >20 U/ml) or by means of the Latex fixation test, and joint score (Thompson joint score, a weighted score including both 38 tender joints and 38 swollen joints, range 0–534 [20]). The Thompson joint score is found to be well correlated with other frequently used joint scores [21].

### Radiographic damage

Radiographs of the hands and feet (posterior–anterior view) were taken once every year and were scored in chronological order according to the modified Sharp/van der Heijde method [22] by two observers who were blinded for clinical features. Differences in total scores per patient of more than 25% were discussed until agreement was reached. The intraclass correlation coefficient between two sets of scores was 0.98, indicating excellent agreement [4]. The total radiographic damage score is the sum of the joint space narrowing and the erosion scores of both hands and feet, ranging between 0 and 448 (a higher score indicating more damage).

### Biomarker analyses

Four biomarkers were evaluated — namely C1,2C, C2C, CS846-epitope, and CPII. All assays are described and were used as recommended by the manufacturer (Ibex, Montreal, Canada).

The C1,2C competitive inhibition ELISA assay (a cartilage/skin/bone collagen breakdown assay) measures the carboxy terminus of the primary cleavage site (Col 2–3/4C_Short _epitope) generated in type I and type II collagens by collagenases [11].

The C2C competitive inhibition ELISA (a cartilage breakdown-specific assay) measures a related carboxyterminal neoepitope created by the cleavage of only type II collagen by collagenases. This longer neoepitope is specific for type II collagen [10].

The CS846 ELISA assay measures an epitope on chondroitin sulfate chains of the largest cartilage proteoglycan aggrecan [14]. Differences in the serum epitope content have previously been observed in patients with different rates of RA progression [13]. An ELISA format was used in the present study, whereas a radioimmunoassay format was used in previous studies [13,14].

Another ELISA assay was used to measure the synthesis of type II procollagen by detection of the carboxy propeptide (CPII), which is cleaved from type II procollagen following release of newly synthesized procollagen into the matrix [15]. A radioimmunoassay format was employed in previous studies [13,15].

The intraday (*n *= 20) and interday (*n *= 200) coefficients of variance for each biomarker were, respectively: for C2C, 10–17% and 14%; for C1,2C, 5–14% and 13%; for CS846-epitope, 4–12% and 12%; and for CPII, 11–18% and 16%. The interassay coefficients of variance for all the assays determined for 30 masked pairs were in the range of 6.4–11.5% (KD Brandt, SA Mazzuca, T Lobanok, AR Poole, unpublished data).

Because combinations of markers measuring the balance between different processes such as the synthesis and degradation or differential degradation of cartilage collagen might provide additional information, the following three ratios of biomarkers, and the reverse of these ratios, were calculated: C1,2C/C2C ratio, C2C/CPII ratio, and (C1,2C/C2C)/CPII ratio.

### Statistical analysis

Statistical analyses were performed using the SPSS statistical package ver. 11.5 (2000) (SPSS Institute, Chicago) and SAS statistical package ver. 4.16 (1994) (SAS Institute, Cary, NC).

#### Clinical variables

Spearman rank correlations were calculated to determine possible correlations between biomarker levels and (several) outcomes of disease activity at year 1. Furthermore, we compared biomarker values at year 1 for women versus men, and for patients with a positive rheumatoid factor test versus patients with a negative rheumatoid factor test at 1 year (Mann–Whitney *U *test). We also calculated the correlation between the mean within-patient biomarker concentration over time and the average disease activity over time, calculated as the area under the curve standardized to time (AUC).

#### Associations with radiological damage

Two groups of patients were distinguished to assess whether biomarker levels could discriminate between those patients with slow versus rapid radiographic progression. The annual progression rate (units/year) was defined as the difference between the radiographic damage score measured at 4 years and the score measured at 1 year, divided by three. If no radiographs were obtained at one of these two points, the progression rate was calculated based on available scores. First, the median biomarker levels of patients with slow radiographic progression (lowest tertile of annual progression rate) were compared with biomarker levels of patients with rapid radiographic progression (highest tertile of mean annual progression rate), separately at each assessment point in time. Levels of biomarker concentrations are shown as the median (IQ_0.25–0.75_), and the statistical significance of the differences was tested by a nonparametric Mann–Whitney *U *test (*P *< 0.05).

#### Biomarker ability to predict progression of radiographic damage

Most importantly, with respect to clinical relevance of biomarker evaluation, we determined whether the biomarker value assessed 1 year after disease onset could predict radiological progression during the subsequent years, where the individual patients' rate of progression was estimated as the slope of radiological scores over time (years). Multivariable generalized estimated equation (GEE) analyses, an extension of multiple linear regression for longitudinal repeated-measurements data [23], were performed separately for log-transformed values of total radiographic damage score, erosion score, and narrowing score.

Each GEE model estimated three regression coefficients related to the association between a given biomarker and progression of the damage score representing the effects of, respectively, the biomarker value at year 1, the time since year 1, and the biomarker-by-time interaction. Notice that the first coefficient represents a variable fixed in time, whereas the latter two coefficients are assigned to time-dependent variables whose values change over time. The hypothesis that the progression rate is associated with the one year biomarker value was tested by assessing the statistical significance of the interaction coefficient. Its value of the interaction coefficient measured the strength of this relation. This analysis was repeated after adjusting for patients' gender and age one year after inclusion.

Along with the long-term predictive value, we also evaluated the short-term predictive value of each biomarker for radiological progression during the subsequent year. GEE generalization of the multivariable linear regression was again used. The dependent variable was defined as the difference between the log-transformed radiological scores at year (*i *+ 1) versus the scores year (*i*) using the differences of year 2 minus year 1, of year 3 minus year 2, and of year 4 minus year 3; each subject therefore contributed to up to three differences. The independent variables included the biomarker score at year *i *as well as age and gender.

## Results

### Patients

The study included 87 patients with RA. Serum samples were available for 85 patients at one year, for 79 patients at two years, for 72 patients at three years, and for 77 patients at four years. Demographic and clinical characteristics of these patients 1 year after diagnosis are summarized in Table 1. The mean age was 58 years, and 63% of the participants were female.

### Clinical and demographic variables and biomarkers

One year after diagnosis we did not find statistically significant differences in median (IQ_0.25–0.75_) biomarker values between women and men, while age was not correlated with any of the four biomarkers. No significant differences were also found in biomarker levels between the four assigned treatment groups. All median biomarker concentrations, except for CPII (174 versus 173, *P *= 0.414), were statistically significantly higher for patients with a rheumatoid factor positive test at 1 year (*n *= 62) compared with patients with a negative test (*n *= 24): 136 versus 111 for C2C (*P *= 0.012); 535 versus 371 for C1,2C (*P *= 0.009), and 64 versus 47 for CS846-epitope (*P *= 0.022). Of all other disease-related variables at year 1, the ESR was positively correlated with C2C (*r *= 0.230, *P *= 0.035) and with C1,2C (*r *= 0.256, *P *= 0.019), and almost statistically significantly correlated with CS846-epitope (*r *= 0.215, *P *= 0.051), but not with CPII (*r *= 0.122, *P *= 0.273). The results of these correlations at 1 year remained similar when taking into account the AUC for clinical variables and the mean biomarker concentrations over time. The AUC ESR correlated with C2C (*r *= 0.226, *P *= 0.035), with C1,2C (*r *= 0.261, *P *= 0.015), and with CS846-epitope (*r *= 0.238, *P *= 0.028), but not with CPII (*r *= 0.127, *P *= 0.245). In addition, of all other clinical variables measured, the Thompson joint score at year 1 was only correlated with C2C, although no such correlation was observed for the AUC Thompson score.

It was shown in previous studies that the body mass index correlated with biomarker values [24,25]. To determine whether the body mass index could possibly be a confounding factor, we included the body mass index in our analyses of 74 patients for whom both height and weight were measured at diagnosis. No association for body mass index and biomarker concentrations was found (all *P *values above 0.05); we therefore did not control for body mass index in further analyses.

### Associations between biomarker levels and radiographic damage

**Figure 1:**
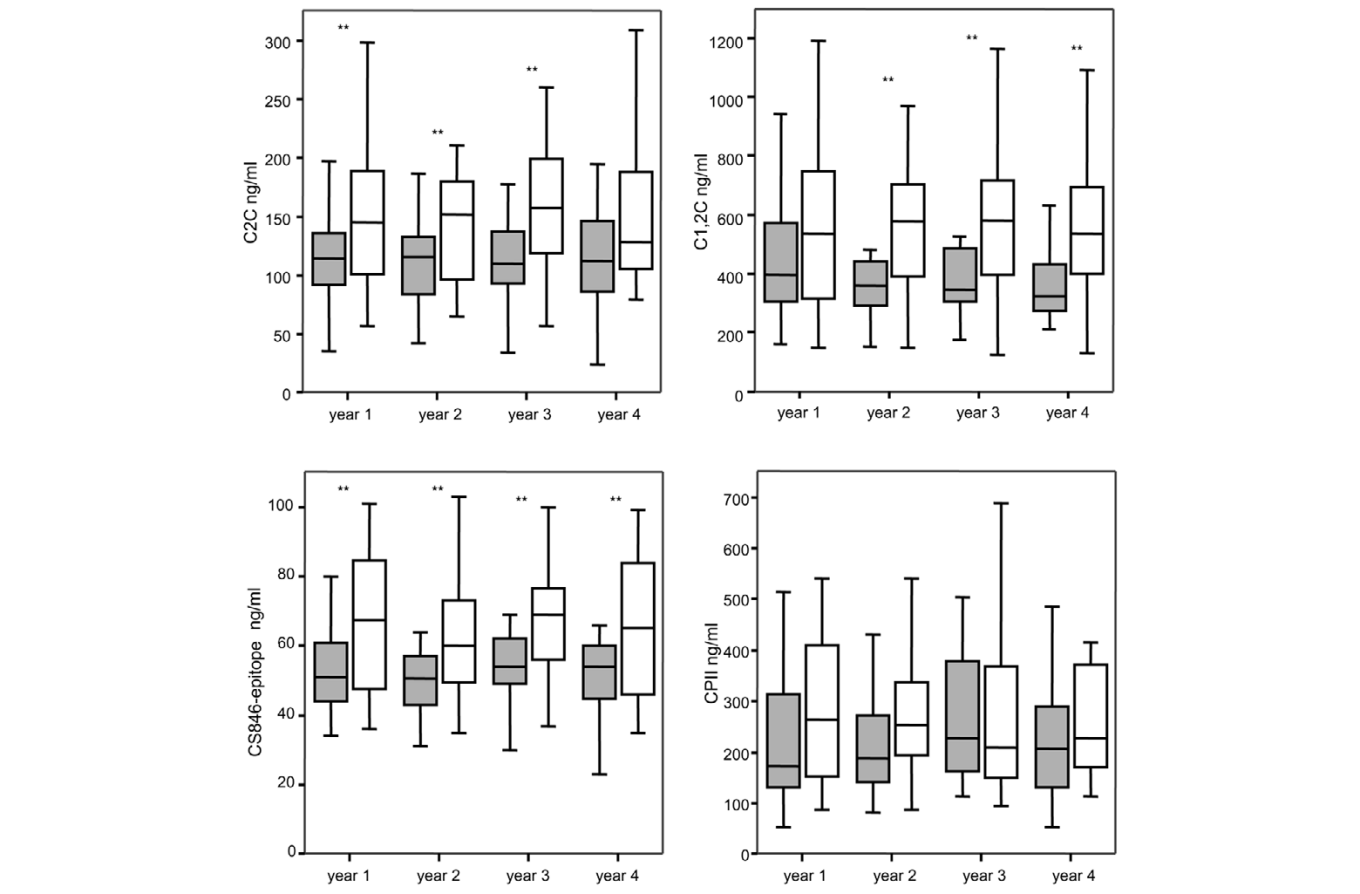
Boxplots of biomarkers levels during follow-up (1–4 years) for patients with slow radiographic progression (<2.33 Sharp/van der Heijde units per year; shaded boxplot) versus patients with rapid radiographic progression (>7.35 Sharp/van der Heijde units per year; white boxplot) based on the 33rd and 66th percentile of annual radiographic progression. Boxplots show the median value and (IQ_0.25–0.75_). Lines outside boxes represent the 10th and 90th percentiles. **Statistically significant (*P *< 0.05) difference between the group of patients with rapid radiographic progression versus the group of patients with slow radiographic progression. C2C, marker for degradation of type II collagen in cartilage; C1,2C, marker for degradation of type I collagen and type II collagen in cartilage; CPII, marker for synthesis of the procollagen of type II collagen cartilage; CS846-epitope, marker for aggrecan turnover in cartilage.

Patients with rapid radiographic progression (highest tertile, >7.35 units per year) had higher median C2C levels, C1,2C levels, and CS846-epitope levels at almost all time points, except for C2C at year 4 and for C1,2C at year 1, than patients with slow radiographic progression (lowest tertile, <2.33 units per year) (Figure 1). In contrast, the median values of CPII were unaffected by radiographic progression at all annual assessment points (Figure 1).

Differences were found for the median radiographic progression rates at 1 year when the highest tertile of each biomarker value was compared with the remainder of the population at year 1. Statistically significant differences were found for C2C (7.8 versus 3.5, *P *= 0.030) and for CS846-epitope (7.5 versus 2.7, *P *= 0.06). However, the median progression rate was not statistically significant for C1,2C (5.5 versus 3.8, *P *= 0.128) and for CPII (6.6 versus 3.7, *P *= 0.127) at year 1.

### Assessing ability of biomarkers to predict progression of radiographic damage

Table 2 presents the results of the GEE analyses of the repeated measurements of the radiological scores (specifically, for each combination of one of the four biomarkers and one of the three damage scores). A statistically significant interaction was found for C2C, for C1,2C, and for CS846-epitope. This means that the progression rate is affected by the value of the biomarker 1 year after disease onset. In such a case, the two progression rate columns of Table 2 show how much the estimated annual rates of change in the damage score increase due to a change of one standard deviation in the biomarker value. For example, there is a statistical significant interaction between time and C2C (*P *= 0.03). For patients with the mean C2C value, the total radiographic damage score increases by 29% per year; whereas for patients with C2C one standard deviation above the mean, the average annual increase is as high as 35%. The relative percentage increase of the progression rate of the radiographic damage score per one standard deviation increase in C2C is therefore 21%.

If the interaction is nonsignificant (*P *> 0.05) there is no evidence that the progression rate varies with the baseline biomarker value. The association between a higher C2C/CPII ratio and faster progression was marginally significant (*P *= 0.055). No other associations were found with the predefined ratios of biomarkers and radiographic progression scores. After a subdivision of the total radiographic damage score into narrowing and the erosion score, higher C2C remained statistically significantly associated with a faster progression of the narrowing score (*P *= 0.019) and only an elevated CS846-epitope was associated with a faster progression of the erosion score (*P *= 0.014). Adjustment by age and sex did not change these results (data not shown).

In addition to the long-term predictive value of the biomarker levels measured at year 1, we also focused on the putative association between a biomarker value observed at a given visit and the progression during the subsequent year. Higher C2C values were associated with a statistically significantly increased progression of radiographic damage during the next year. An increase in the current C2C value by one standard deviation (for example, by 62 units) was associated with a 5% relative increase in the value in the following year of the radiographic damage score among subjects who have the same current score. Moreover, higher current values of both C1,2C and CS846-epitope and lower more recent values of the CPII/C2C ratio were all marginally associated with an increased progression of radiological damage in the subsequent year. Associations of recent biomarkers with progression of erosion or the narrowing score was only apparent for C2C, which was statistically significantly associated with the progression of the joint space narrowing score over the following year (for example, a relative increase of 6.4% in the joint space narrowing score during the next year caused by a one standard deviation increase in the C2C value.

## Discussion

The search for markers to predict radiographic joint damage is important because differences in clinical parameters such as inflammation might not always correlate with radiographic outcome reflective of skeletal damage. Prognostic indicators of joint damage are sought that identify the processes that result in joint damage and which may predict the progression of joint damage. Radiographs only record the outcome, namely joint damage. Hence measurement of a product of the process resulting in joint damage may be of value in predicting such an outcome. A number of assays have recently become available to measure the degradation and repair of bone and cartilage. These biomarkers have been tested mainly in patients with osteoarthritis [8,9], and to a lesser extent in patients with RA [7,12]. Only urinary CTXI [26] and CTXII [12], markers of the degradation of type I collagen and type II collagen, respectively, have presently been demonstrated to be predictive of joint damage in RA.

In the present study, a set of other cartilage biomarkers of joint damage and turnover was evaluated. We measured not only the degradation (C1,2C and C2C), but also the turnover and synthesis (CS846-epitope and CPII, respectively) of cartilage. Interestingly, it was the biomarkers for degradation of collagen (C2C and C1,2C) and turnover of aggrecan (CS846-epitope), rather than for the synthesis of cartilage collagen (CPII), that were significantly elevated in patients with rapid radiographic progression when compared with patients with slow progression. It thus seems that the development of radiographic damage during the first years after diagnosis is more a reflection of increased degradation of collagen and enhanced turnover of proteoglycans rather than a lack of synthesis of cartilage collagen. This lack of association between CPII and radiographic damage corroborates previous findings in a cohort of RA patients [13].

More importantly, C2C, C1,2C and CS846-epitope measured at one year were each predictors of radiographic damage during the subsequent years. Furthermore, C2C levels measured at a given year predicted radiographic progression during the subsequent year. These results are in perfect agreement with the studies showing urinary CTXII, a type II collagen degradation marker for cartilage loss, to be associated with concurrent severity of radiographic damage and to predict its future progression in RA [12]. In our analyses, as might be anticipated, it was specifically joint space narrowing (for example, cartilage loss), and with that type II collagen loss, that could be predicted by serum C2C levels – not only each year, but also over the period of study. This no doubt reflects the fact that C2C is a specific marker for cleavage of type II collagen, which is primarily present in hyaline cartilage and the intervertebral discs with relatively very small amounts present elsewhere in other tissues, such as in entheses and in the vitreous of the eye. This was not true for aggrecan turnover (CS846-epitope), however, which was only associated with total radiographic damage score and with the erosion score, and not with narrowing score as one would expect.

Another interesting finding of this study is that biomarker concentrations differed extensively between patients but mean concentrations remained remarkably stable over time for all four biomarkers, suggesting that the process of cartilage degradation follows a continuous stable course after 1 year of diagnosis of RA. Interestingly, we have also found a linear progression of radiographic damage in our cohort during the first years after diagnosis [4].

In this study, we further determined whether biomarker levels correlated with clinical parameters or were associated with demographic characteristics. Only the ESR, both at one year and over time, was correlated with C2C, with C1,2C, and with CS846-epitope concentrations. Also, patients who had a positive rheumatoid factor test at 1 year had significantly higher levels of C2C, of C1,2C, and of CS846-epitope. Our primary goal was to study the pathogenic role of biomarkers alone in relation to radiographic progression, and we therefore did not correct for the ESR and rheumatoid factor. When biomarkers levels at 1 year were adjusted for the ESR and rheumatoid factor obtained at 1 year, the predictive ability for total radiographic progression over 4 years decreased, but was still evident for C2C (*P *= 0.065), for C1,2C (*P *= 0.026), and for CS846-epitope (*P *= 0.017).

We used a random set of samples because we wanted a representative RA population. We therefore think that the results could improve if we had sorted samples based on extreme high values of radiographic damage score versus patients with hardly any damage. In this study we did not want to determine the influence of treatment on change of biomarker levels, but rather the association between biomarker levels over the years with radiographic damage during follow-up and the predictive ability of biomarker levels for radiographic damage. We therefore started sera samples analyses one year after the start of treatment since changes in disease activity are considerable in the first year after initiating treatment, varying significantly between patients. For CTX-II and CTX-I it was found that the CTX-I values decreased during the first six months because of treatment, but after 1 year both CTX-I and CTX-II values were similar to baseline values [27]. More longitudinal studies are necessary to better determine the clinical value of the biomarkers used in our investigation. In these studies the predictive ability of biomarkers associated with cartilage breakdown measured at diagnosis, and before treatment, should also be evaluated. In general, the identification of biomarkers that can be used as a prognostic tools in daily practice to predict the onset and progression of joint damage remains the goal of these studies in RA, and especially in osteoarthritis [28], where the ability to predict progression of cartilage destruction and outcome is perhaps of even greater importance.

## Conclusion

This study shows that biomarkers of cartilage collagen breakdown (C2C and C1,2C) and proteoglycan turnover (CS846-epitope), but not biomarkers of synthesis (CPII), are related to specific joint space narrowing and erosions in RA. Specifically, C2C (the marker for collagen type II damage) could predict subsequent short-term as well as long-term radiographic damage in RA, and more specifically joint space width narrowing.

## Abbreviations

AUC = area under the curve; C1,2C = marker for degradation of type I collagen and type II collagen in cartilage; C2C = marker for degradation of type II collagen in cartilage; CPII = marker for synthesis of the procollagen of type II collagen cartilage; CS846-epitope = marker for aggrecan turnover in cartilage; ELISA = enzyme-linked immunosorbent assay; ESR = erythrocyte sedimentation rate; GEE = generalized estimated equation; IQ_0.25–0.75 _= Interquartile range (25^th^–75^th ^percentile); RA = rheumatoid arthritis.

## Competing interests

AR Poole consultant to IBEX.

LE King employee at IBEX.

## Authors' contributions

SMMV contributed to the conception and design of the study, collected data, scored the radiographs, performed biomarker analyses and statistical analyses, and helped to draft the manuscript. ARP developed the biomarker assays, contributed to the conception and design of the study, and contributed to drafting the manuscript. MI developed the biomarker assays, contributed to the conception and design study, and performed biomarker analyses. LEK developed the biomarkers assays, contributed to the conception and design of the study, and helped to draft the manuscript. MA contributed to the conception and design of the study, performed statistical analyses, and contributed to drafting the manuscript. DMH recruited patients, assessed clinical variables, scored radiographic damage, and contributed to drafting the manuscript. JWJB recruited patients, assessed clinical variables, contributed to the conception and design of the study, and contributed to drafting the manuscript. FPJGL contributed to the conception and design of the study, and contributed to drafting the manuscript.

## Figures and Tables

**Table 1:** Patient characteristics, disease-modifying antirheumatic drug (DMARD) use, and biomarker concentrations 1 year after diagnosis

Age (years)	58 ± 13
Gender, women (%)	63
Rheumatoid factor, positive (%)	68
Erythrocyte sedimentation rate (mm/h^1st^)	25 ± 25
Joint score, Thompson	52 ± 77
Morning stiffness (min)	44 ± 88
Visual analogue scale pain (mm)	24 ± 25
Visual analogue scale general well-being (mm)	31 ± 26
Functional disability score, Health Assessment Questionnaire	0.96 ± 0.70
Radiographic damage, erosions	7.2 ± 8.3
Radiographic damage, narrowing	3.7 ± 4.1
Total radiographic damage score, Sharp/van der Heijde	10.9 ± 11.2
DMARD use [number (%)]	
No DMARD	6 (7)
Intramuscular gold	19 (22)
Methotrexate	26 (30)
Hydroxychloroquine	21 (24)
D-penicillamine	7 (8)
Sulfasalazine	4 (5)
Imuran	1 (1)
Auranofin	3 (3)
Biomarker concentrations	
C2C (ng/ml)	130 ± 62
C1,2C (ng/ml)	514 ± 287
CS846-epitope (ng/ml)	61 ± 22
CPII (ng/ml)	242 ± 180

Values presented as the mean ± standard deviation for continuous variables and percentages for categorical variables. Rheumatoid factor positive, patients testing positive either at diagnosis or 1 year. C2C, a marker for degradation of type II collagen in cartilage; C1,2C, a marker for degradation of type I and type II collagen in cartilage; CS846-epitope, a marker for aggrecan turnover in cartilage; CPII, a marker for synthesis of the pro-collagen of type II collagen cartilage.

**Table 2:** Association between biomarker values measured at year 1 and subsequent radiographic progression rates

Biomarker	*P *value for interaction^a^	Estimated annual progression rate	
		
		Mean biomarker (%)^b^	Mean + one standard deviation (%)^c^
C2C			
Damage	0.030*	29	35
Erosion	0.156	24	27
Narrowing	0.019*	31	40
C1,2C			
Damage	0.033*	26	30
Erosion	0.195	25	29
Narrowing	0.271	32	36
CPII			
Damage	0.777	28	28
Erosion	0.816	25	25
Narrowing	0.136	33	41
CS846-epitope			
Damage	0.039*	28	34
Erosion	0.014*	27	36
Narrowing	0.476	32	35

For each biomarker, the results of the multivariable generalized estimated equation regression model for longitudinal analyses between biomarker value at year 1 after inclusion and subsequent (repeated over time) values of the log-transformed progression score are presented. C2C, marker for degradation of type II collagen in cartilage; C1,2C, marker for degradation of type I collagen and type II collagen in cartilage; CPII, marker for synthesis of the procollagen of type II collagen cartilage; CS846-epitope, marker for aggrecan turnover in cartilage. ^a^*P *< 0.05 indicates that the change in the rate of progression depends significantly on the biomarker value at year 1; *interaction statistically significant (*P *< 0.05). ^b^Percentage increase of radiographic damage score per year for patients with a mean value of biomarker 1 year after disease onset. ^c^Percentage increase in radiographic damage scores per year for patients with a value one standard deviation above the mean biomarker value 1 year after disease onset.
